# The Contribution of Liver Sinusoidal Endothelial Cells to Clearance of Therapeutic Antibody

**DOI:** 10.3389/fphys.2021.753833

**Published:** 2022-01-14

**Authors:** Bethany H. James, Pantelitsa Papakyriacou, Matthew J. Gardener, Louise Gliddon, Christopher J. Weston, Patricia F. Lalor

**Affiliations:** ^1^Centre for Liver and Gastroenterology Research and National Institute for Health Research (NIHR) Birmingham Biomedical Research Centre, Institute of Immunology and Immunotherapy, University of Birmingham, Birmingham, United Kingdom; ^2^Antibody Pharmacology, Biopharm Discovery, Glaxo Smith Kline Research and Development, Stevenage, United Kingdom

**Keywords:** liver, endothelium, antibody, therapy, disease

## Abstract

Many chronic inflammatory diseases are treated by administration of “biological” therapies in terms of fully human and humanized monoclonal antibodies or Fc fusion proteins. These tools have widespread efficacy and are favored because they generally exhibit high specificity for target with a low toxicity. However, the design of clinically applicable humanized antibodies is complicated by the need to circumvent normal antibody clearance mechanisms to maintain therapeutic dosing, whilst avoiding development of off target antibody dependent cellular toxicity. Classically, professional phagocytic immune cells are responsible for scavenging and clearance of antibody *via* interactions with the Fc portion. Immune cells such as macrophages, monocytes, and neutrophils express Fc receptor subsets, such as the FcγR that can then clear immune complexes. Another, the neonatal Fc receptor (FcRn) is key to clearance of IgG *in vivo* and serum half-life of antibody is explicitly linked to function of this receptor. The liver is a site of significant expression of FcRn and indeed several hepatic cell populations including Kupffer cells and liver sinusoidal endothelial cells (LSEC), play key roles in antibody clearance. This combined with the fact that the liver is a highly perfused organ with a relatively permissive microcirculation means that hepatic binding of antibody has a significant effect on pharmacokinetics of clearance. Liver disease can alter systemic distribution or pharmacokinetics of antibody-based therapies and impact on clinical effectiveness, however, few studies document the changes in key membrane receptors involved in antibody clearance across the spectrum of liver disease. Similarly, the individual contribution of LSEC scavenger receptors to antibody clearance in a healthy or chronically diseased organ is not well characterized. This is an important omission since pharmacokinetic studies of antibody distribution are often based on studies in healthy individuals and thus may not reflect the picture in an aging or chronically diseased population. Therefore, in this review we consider the expression and function of key antibody-binding receptors on LSEC, and the features of therapeutic antibodies which may accentuate clearance by the liver. We then discuss the implications of this for the design and utility of monoclonal antibody-based therapies.

## Introduction

### The Growing Importance of Therapeutic Antibodies

Monoclonal antibody-based therapies for a variety of conditions have been available since the late 1980s. Therapeutic antibodies are biopharmaceuticals that recognize and bind to a specific antigen leading to either activation or inhibition of downstream biological pathways. Monoclonal antibodies (mAbs) are the most common clinical tool and represent the leading treatment modality for diseases ranging from inflammatory and autoimmune disease to cancer. Upon recognition of cognate antigen they either trigger an antibody mediated cellular cytotoxic (ADCC) and/or a complement-dependent cytotoxic (CDC) effector response, or act to neutralize the intended target antigen. Antibodies are large molecules, which generally don’t interact with transport molecules or detoxification enzymes, exhibit ion channel-related complications or cause immunogenicity. Thus antibody-based therapeutics tend to be potent and well tolerated (Catapano and Papadopoulos, 2013). Only three antibodies were approved by the FDA in 2013 and four in 2014, whereas as of December 2019 a total of 79 mAbs have met approval standards with over 500 currently undergoing clinical trials around the world (Kaplon et al., 2020). Hence the global therapeutic antibody market is predicted to generate over $300 billion by 2025 (Lu et al., 2020).

However, adverse effects post-treatment are not uncommon, and often relate to the pathway being targeted or the mode of action of the drug itself. Importantly problems and adverse events are not always predicted by preclinical screening strategies. Toxicity or adverse events may relate to biological function of the target molecule [e.g., minor bleeds in patients treated with anti-platelet agents such as abciximab (Tamhane and Gurm, 2008)] or interaction with off-target tissues. Less specific toxicity can also be explained by hypersensitivity responses to immunogenic “non” human elements of therapeutics. When designing a new antibody-based therapy there is also a need to minimize interactions with non-target molecules and tissues other than the therapeutic target. These issues can be resolved by careful engineering of antibody to reduce immunogenicity, maximize efficacy, and minimize clearance. Similarly, choice of administration route has an impact on its efficacy and clearance. Intravenous administration rapidly delivers 100% of antibody into the systemic circulation and generates high plasma concentrations, but increases the potential for off target exposure, hypersensitivity reactions and the cost of in-house treatment. In contrast, sub-cutaneous and intra-muscular administration deliver antibody *via* the lymphatic system. Here formulation, injection volume and physical factors such as age and weight of the patient (Richter et al., 2012; Richter and Jacobsen, 2014) can impact on bioavailability. Antibodies destined for use in chronic conditions need to have the longest possible half-life and minimal clearance rates to support a favorable administration strategy and ensure dosing frequency is not prohibitive. Importantly preclinical pharmacokinetic testing of new reagents in a disease specific model is vital to ensure patient demographics for likely clinical use are best represented. In this article we will consider the underestimated role of the liver, and specifically the sinusoidal endothelial cells in antibody clearance. We also consider strategies that could be utilized to minimize hepatic clearance, and the impact of age or chronic disease on endothelial: antibody interactions. We begin with a review of therapeutic antibody generation and structure before considering implications for hepatic targeting and explanations for reported adverse events in clinical use.

### Generation of Antibodies for Therapeutic Use

**FIGURE 1:**
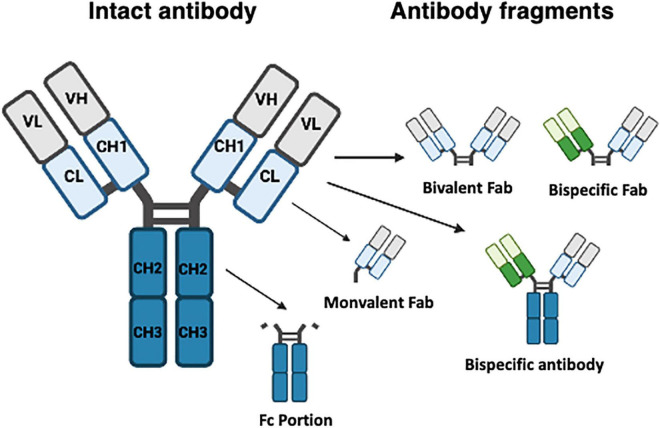
Typical structure of monoclonal and bispecific antibodies. Monoclonal antibodies (left structure) are composed of four polypeptide chains, two light (L) and two heavy (H), each both a Fab fragment and an Fc region (blue) joined by a hinge section to create a Y-shaped structure. The Fab fragment which recognizes antigen is composed of constant (C) and variable (V) domains which make up the antigen binding site. Specific fragments are also shown. Fab fragments can be bivalent or monovalent, and engineered bispecific antibodies can contain or lack an Fc portion.

Therapeutic mAbs have similar structure to endogenous immunoglobulin, i.e., four polypeptide chains, two light and two heavy, each with both a Fab fragment and an Fc region. These form a complex Y-shaped structure (see Figure 1). The Fab fragment is composed of one constant region and one variable domain which make up the antigen binding site. The Fc region at the tail end of the antibody binds to elements of the immune system such as complement components and surface receptors known as Fc receptors (FcRs). Historically, man-made antibodies were generated using the hybridoma technique (Kohler et al., 1976) to generate murine monoclonal reagents as exemplified by OKT3 (Kung et al., 1979). This murine antibody targeting human CD3 antigen on T cells was widely used in immunotherapeutic contexts including management of allograft rejection. However, it has since been withdrawn due to side effects and generation of host anti-murine antibodies which reduced efficacy (Sgro, 1995). Subsequently, the disadvantages of murine mAbs were partially overcome by generation of chimeric antibodies. Here recombinant DNA technology was used to generate hybridized reagents consisting of the variable region from a mouse antibody fused to a human antibody constant region. This reduced the potential for the generation of anti-murine antibodies. The first chimeric mAb approved by the FDA, abciximab (Lu et al., 2020) is a Fab fragment antagonist to glycoprotein IIb/IIIa receptor used to inhibit platelet aggregation. This was soon followed by, the first full length IgG chimeric antibody “rituximab,” an anti-CD20 antibody widely used as an immune modifier (Maloney et al., 1997). To further reduce the risk of immunogenicity, the residual proportion of mouse antibody has been further diminished by the advent of complementarity determining region (CDR) grafting approaches (Riechmann et al., 1988; Tsurushita et al., 2005). Despite the increased proportion of human sequence within such antibodies, adverse reactions still occurred (Nechansky, 2010).

This led to a drive to produce fully humanized reagents through application of technologies such as phage or yeast display of antibody peptide libraries (Smith, 1985; McCafferty et al., 1990). This method is rapid and robust with libraries containing 1 × 10^10^ antibody fragments available and is now considered the gold standard for recombinant antibody production. The anti-TNF antibody Adalimumab was generated using this approach and is currently one of the best-selling therapeutics in the world, generating $20 billion in 2018 (Kempeni, 1999; Lu et al., 2020). Similarly, immunization of transgenic rodents to generate fully humanized antibodies is significant. Here the mouse IgG gene repertoire is replaced with human counterparts leading to development of transgenic lines (Lonberg et al., 1994; Mendez et al., 1997) such as the Xeno-mouse. The huge potential of this technology is exemplified by panitumumab, the first Xeno-mouse reagent to gain FDA approval. This fully human IgG_2_ EGFR antibody is used in therapy for metastatic colorectal cancer (Jakobovits et al., 2007). Currently 19 approved mAbs have been developed using such transgenic mice. This method is advantageous as there is often no requirement for an affinity maturation step for targets with high affinity, and full-length IgG antibodies are made. However, if the antigen being used to immunize is particularly toxic then phage display is the preferred technique. To date human and humanized mAbs are the dominant format of therapeutic antibodies accounting for, respectively, 51 and 35% of all mAbs currently in clinical use (Lu et al., 2020).

Whilst traditional monoclonal antibodies bind to a single antigen, bispecific tools have been engineered to improve targeting [increase the efficacy of immune: target cell or receptor:ligand interactions (Kang and Lee, 2021)] and exhibit favorable tissue penetration. Different formats exist and each has its own advantages and challenges. Fragment based bispecific antibodies (BsAb) lack a Fc region but still contain two independent antigen binding domains. As there is no Fc region present, these BsAb are considerably smaller than traditional mAbs allowing them to penetrate tissues easily. A good example of this approach is blinatumomab used in treatment of lymphoblastic leukemia (Kantarjian et al., 2017). This antibody combines two antigen receptor epitopes to recognize CD3+ effector T cells and CD19+ B cells to stimulate recognition and elimination of B cell blasts. Although effective at improving survival, this approach is not without adverse events including elevation in liver enzymes (Kantarjian et al., 2017). The other formulation is the full-length IgG-like asymmetric BsAb (Fc-based BsAbs, or BsMabs) which retain an Fc portion. Mosunetuzumab used in treatment of leukemia exemplifies this approach again targeting both a B cell epitope (CD20) and CD3 (Schuster, 2021), and also bears a Fc domain engineered to minimize FcγR and complement binding. However, if a strong immune response is required, intact Fc regions facilitate interactions with FcR and C1q. The small size and dual antigen specificity of such bispecific reagents places a target cell in close proximity to the effector cells resulting in a more effective response than more traditional mAbs. Hence such forms of BsAb have low therapeutic concentrations and short half-life, (Wang et al., 2019) which can meant that frequent infusions are required possibly increasing potential for off target effects. More recently there have been attempts to improve specificity of targeting by using gene therapy approaches to drive cell specific expression of bispecific antibodies at the site of need. This is particularly attractive if hepatospecific targeting is required, given the high phagocytic activities and ready absorbance of liposomes and nanosomes within the liver. This approach is elegantly exemplified by the work of Kruse et al. (2017) who generated hepatitis B Ag : CD3 specific bispecific antibodies with antiviral efficacy *in vivo* (Kruse et al., 2017).

### A Focus on Fc Receptors and Mechanisms of Antibody Uptake and Clearance

Highly charged cationic molecules like antibodies with poor pharmacokinetic profiles are cleared reasonably quickly (Haraya et al., 2019) and evidence suggests that this clearance takes place in highly vascularized organs like the liver and spleen (Li et al., 2014). The liver in particular is a major site for internalization and catabolic clearance of therapeutic antibodies as they are typically too large for renal elimination. This is facilitated in part by an impressive scavenging system. Cells of the hepatic reticuloendothelial system express many receptors that can bind and internalize antibodies either by target mediated clearance or *via* non-specific uptake. As noted above, Fc receptors on a cell surface generally recognize the Fc portion of antibody and as a consequence activate and modulate immune responses or clear immune complexes. This could take the form of destruction of an opsonized target cell or the activation/regulation of cellular effector responses. However, exaggerated antibody-dependent autoimmune and hypersensitivity responses and circulating therapeutic antibody pharmacokinetics are also impacted by the action of these receptors (Hogarth and Pietersz, 2012). In the context of antibody-based therapies, interaction with FcR is important for specific targeting of an immune response. The Fc gamma receptor (FcγR) family of proteins consists of six FcγRs in humans which include FcγR1 (CD64), FcγRIIa,b and c (CD32a-c) and FcγRIIIa and b (CD16a and b) (Brooks et al., 1989). Each has a slightly different cellular distribution and affinity for IgG (Hogarth and Pietersz, 2012). Human IgG1 and 3 bind more effectively to FcγRs than IgG2 and 4 (Schwab et al., 2015) but IgG1 antibodies are still the most commonly used for therapies (Lucas et al., 2018). Clustering of antibody and target antigen may be enhanced by binding to FcγRIIb (Stopforth et al., 2016). In contrast, internalization, and catabolism of antibodies *via* FcγR may be particularly important for antibodies with circulating soluble antigens or which form large immune complexes with target as these tend to bind well to FcRs (Lucas et al., 2018).

Engagement of receptor on immune cells generally induces a cellular response *via* activation of immunoreceptor tyrosine-based activation motif (ITAM) and SRC family kinase activation. In most cases this causes a pro-inflammatory response, but FcγRIIb has inhibitory effects *via* activation of immunoreceptor tyrosine-based activation motif (ITIM) (Hogarth and Pietersz, 2012), despite binding IgG with a relatively low affinity. In B cells this can downregulate signals from the other FcR and cause apoptosis. There are also descriptions of two variants of FcγRIIb (b1 and b2) which have slight differences in the ability to internalize antibody due to variance in structure of the cytoplasmic domain of the receptor (Stopforth et al., 2016). The neonatal Fc receptor (FcRn) seems to be more involved in antigen presentation and IgG recycling within cells. It is expressed by endothelium (Vaccaro et al., 2005), tissue macrophages and Kupffer cells, enterocytes and some epithelial cells (Latvala et al., 2017). It is atypical in that along with binding IgG it also recognizes albumin and plays key roles in transcytosis and recycling of both to maintain circulating concentrations (Pyzik et al., 2019).

**FIGURE 2:**
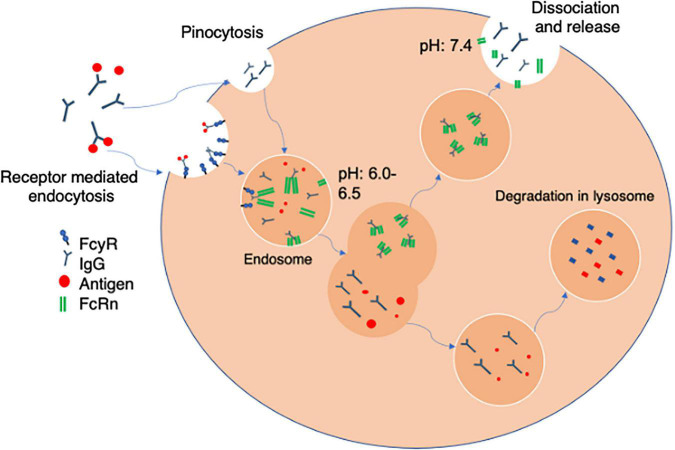
Receptor mediated antibody uptake. The Fc portion of free antibody or antibody bound to soluble antigen to form an immune complex bind to FcγR at the cell surface. Once bound antibody is internalized into an acidified endosome *via* fluid phase pinocytosis. The endosomes contain FcRn which binds *via* the heavy chains in the Fc region in a pH sensitive manner. The FcRn can then recycle bound antibody back to the cell membrane where physiological pH of blood allows uncoupling and release back into the circulation. Alternately mAbs that fail to be recycled by FcRn are either cleared *via* the activation of C1q, and the classical complement pathway or are degraded by proteases present within lysosomes within the cell.

The process for uptake and recycling of antibody is described in Figure 2. Once bound to FcγR a monoclonal antibody is internalized into an endosome. Here they encounter membrane bound FcRn (Roopenian and Akilesh, 2007) which is responsible for the protection of IgG catabolism, recycling the antibody to the surface leading to an increased half-life. This binding is pH dependent and will only occur in acidic endosomes, with a pH at around 6–6.5. FcRn containing vesicles become exposed to an increasing pH gradient until they reach the cell surface and physiological pH. This causes the mAb and FcRn to dissociate and the antibody is then released from the cell and recycled back into circulation. mAbs that fail to be recycled by FcRn are either cleared *via* the activation of C1q, and undergo clearance *via* the classical complement pathway or are degraded by proteases present within lysosomes (Leipold and Prabhu, 2019). Therefore the FcRn is important to spare the mAb from degradation and prolong the half-life (Haraya et al., 2019) potentially reducing therapeutic dosing and frequency. Some studies have suggested that it is FcRn that primarily impacts on pharmacokinetics and that FcγRIIb has little impact on circulating antibody distribution (Abuqayyas et al., 2013). However, it is important to note that some studies with knockout animals deficient in FcγRIIb tested antibodies at concentrations far below therapeutic concentrations. Even in these circumstances there was an increase in liver distribution (albeit variable) even at low dose suggesting that within the liver FcγRIIb may be involved in clearance and degradation of antibody (Abuqayyas et al., 2013). This seems to be particularly important for antibody: antigen complexes which are cleared into liver whilst antigen alone is not (Ljunghusen et al., 1990). Thus, in the next section we describe the function of the hepatic sinusoidal endothelial cells to highlight their potential roles in antibody bioavailability.

### Liver Sinusoidal Endothelial Cell Structure and Function

**FIGURE 3:**
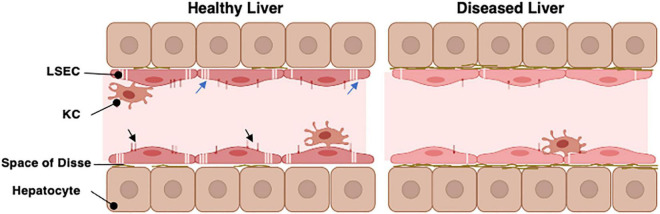
The organization of the hepatic sinusoid. The hepatic sinusoids represent the capillary bed of the liver and are lined by specialized liver sinusoidal endothelial cells (LSEC). These sit above the hepatocyte layer separated only by the Space of Disse which contains minimal basement membrane in a healthy liver. LSEC have specialized pores in their cell surface (the fenestrations, blue arrows) which organize into sieve plates to facilitate direct exchange of materials between the hepatic parenchyma and bloodstream. The LSEC also express unique profiles of cell surface scavenger receptors and Fc receptors (Black arrowheads) which can interact with macromolecules within the slow flowing sinusoidal blood. Kupffer cells (KC) are specialized macrophages which patrol along the sinusoids to fulfil their immune regulatory functions. In chronic disease or aged livers, the nature of the LSEC changes. They lose most of their fenestrations and alter abundance of scavenger and Fc receptors. They also produce a more complex basement membrane. This restricts movement of materials into and out of the parenchyma.

One factor which remains challenging in the development of antibody therapies relates to their pharmacokinetics and clearance in tissue. This alters exposure to target antigen and ultimately efficacy. Distribution within a tissue is impacted upon by movement across the vessel wall and interaction with endothelial cells and macrophages which express the receptors described above. Tissues like the liver which have fenestrated non-continuous endothelial cells, are highly perfused and abundantly vascularized, will have greater exposure to antibody (Datta-Mannan, 2019). The isoelectric point of an antibody appears to particularly influence hepatic clearance, such that engineering of antibody variants with high pI leads to preferential sequestration and clearance by the liver (Ganesan et al., 2012). Transport of antibody from blood into tissue is dependent on local perfusion gradient and key features of the vessel wall such as presence of fenestrated endothelium and basal lamina thickness. Junctional structure is also important with the presence of endothelial cells containing tight junctions limiting access, as is seen in the brain (Tabrizi et al., 2010). Thus, the liver sinusoidal bed presents a particular challenge. Liver sinusoidal endothelial cells (LSEC, Figure 3) which are exposed to both systemic and portal blood are designed to maximize the exchange of useful material from the blood into the liver and vice versa (Shetty et al., 2018). They form part of the hepatic reticuloendothelial system with roles in both the clearance of detrimental pathogens and waste products and the transport of important metabolic products to and from the proximal hepatocytes. These activities are facilitated by the presence of numerous macroscopic pores or “fenestrations,” organized into sieve plates which transverse the full thickness of the endothelial layer allowing transport of lipids and proteins (Hunt et al., 2019) and also medicinal drugs such as lidocaine and paracetamol (Mitchell et al., 2011). Importantly unlike the kidney (Satchell and Braet, 2009) and other organs, the hepatic sinusoidal endothelial fenestrations lack a diaphragm and basal lamina. This, plus the ability of cells to rapidly regulate fenestration diameter and number (O’Reilly et al., 2010; Cogger et al., 2016) further regulates transport.

**FIGURE 4:**
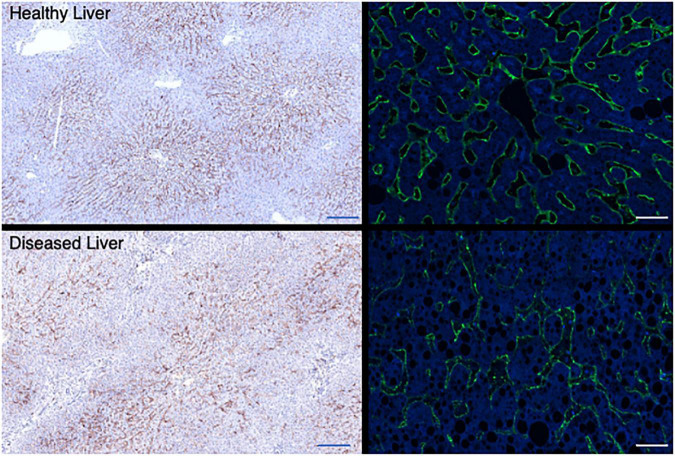
Hepatic sinusoidal endothelial expression of FcγR2b alters in disease. Representative immunochemical (left panels, 10× original magnification Bar is 200 um) and immunofluorescent stains (right panels, 100× original magnification, Bar is 20 um) for FcγR2b on representative examples of healthy (top row) and diseased liver [bottom row, cirrhotic explanted liver from patient with PSC (left) or ALD (right)]. FcγR is localized to the LSEC in both cases, but expression is more intense and consistent across the sinusoid in a healthy context. In explanted cirrhotic human livers some areas of sinusoids lack expression completely.

**FIGURE 5:**
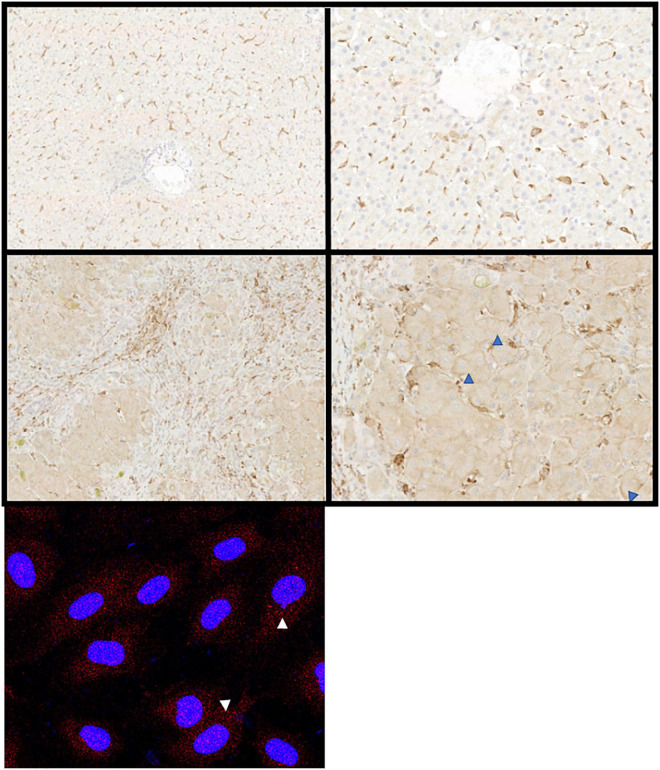
Hepatic expression of FcRn alters in disease. Representative immunochemical (top panels), and immunofluorescent stains (bottom left panel) for FcRn on representative examples of healthy (top row) and diseased liver (middle row) or primary cultures of human LSEC. Both hepatocytes and sinusoidal cells express FcRn but the intensity increases in disease (ALD, middle row). Hepatocellular membrane expression increases as disease progresses (blue arrowheads). Original immunochemical stain images captured at 10× and 50× magnification (left and right panels, respectively). Cultured LSEC express FcRn (red stain) in an intracellular vesicular pattern (white arrows).

Liver sinusoidal endothelial cells also express an unusual complement of scavenger receptors which recognize, bind, and rapidly internalize an enormous diversity of extracellular ligands (Shetty et al., 2018). These are characterized into classes A to J depending on their ligand recognition and structural properties (Patten et al., 2021) and LSEC express receptors in classes SR-B, E, F, G, and H to support clearance of fatty acids, lipids ECM proteins, glycosaminoglycan molecules and apoptotic cells. This significant endocytic capability supports the immune regulation (Knolle and Limmer, 2001), metabolic capacity (Li et al., 2011) and “waste management” (Smedsrod, 2004) functions of the liver. In the context of this article, it is important to note that LSEC also express high levels of FcR under homeostatic conditions. The FcR on LSEC can bind opsonized pathogens and macromolecules to facilitate clearance, with blood-borne immune complexes rapidly cleared from the circulation by both Kupffer cells (KC) and LSEC (Smedsrod, 2004). Although KC may be more efficient at clearing immune complexes, the increased number of LSEC compared to KC within a liver means that their total capacity may be similar (Johansson et al., 2000). Circulating immune complex clearance can cause tissue damage and inflammation in some conditions (Johansson et al., 2000) and thus sinusoidal endothelial cells contribute to the process of clearance *via* the FcR interaction (Johansson et al., 2000). This may be particularly important when the load of circulating IgG is high (Johansson et al., 2000). LSEC have been suggested to express all three of the major Fcγ receptors (Smedsrod, 2004) and it is estimated that up to 75% of all the FcγRIIb within the body is expressed on LSEC (Ganesan et al., 2012). Thus, this abundant receptor expression plays a key role in removal of small immune complexes from blood. We have documented expression in human livers (Figure 4) and confirm that expression is abundant and localized to LSEC in the healthy liver. Expression is maintained in chronic disease (Figure 4) but the distribution is altered in cirrhosis and intensity of staining is reduced, which may suggest an impact on function. FcRn has a more widespread hepatic distribution, described to be present on epithelial cells, endothelium, and immune cell populations (Pyzik et al., 2019) in animal studies. In agreement, our investigation of human liver (Figure 5) confirms intense sinusoidal expression localized to Kupffer cells. Periportal immune cells are also positive with a degree of intracellular staining in hepatocytes. Faint intracellular LSEC staining is confirmed by confocal studies (Figure 5 final panel) on cultured human LSEC. Although historically the role of FcRn LSEC has not been well documented (Skogh et al., 1985), hepatocyte intracellular FcRn (Pyzik et al., 2019) has been linked to clearance and catabolism of antibody and albumin transport. Interestingly we also see intracellular localization in human hepatocytes (Figure 5) with increased peri-membranous distribution in advanced disease (Blue arrows Figure 5). This may reflect a response to hypergammaglobulinemia in cirrhosis and liver disease (Alonso et al., 2012; Cacciola et al., 2018). FcRn also plays roles in the pathology of toxic liver injury. Drugs including paracetamol are transported bound to circulating albumin, and blockade of the interaction between albumin and FcRn reduces hepatotoxicity after paracetamol administration (Pyzik et al., 2017). Interestingly LSEC also express a scavenger receptor lectin, dendritic cell specific ICAM-3 grabbing non-integrin (DC-SIGN) (Lai et al., 2006; Schwab and Nimmerjahn, 2013) which has been demonstrated to be a coreceptor for some viruses (Gramberg et al., 2007). This receptor also bind intravenously administered therapeutic Immunoglobulin (IVIg) (Hogarth and Pietersz, 2012; Schwab and Nimmerjahn, 2013), upregulates expression of FcγRIIb and protects against immune-complex mediated disease (Anthony et al., 2011).

All the evidence above suggests that in a healthy liver, the LSEC are armed with key receptors and endocytic machinery to bind and transport antibody and immune complexes. There is functional evidence to support this. For example, studies of clearance of Bispecific antibodies in cynomolgus monkeys suggest a role for both macrophages and LSEC in clearance (Datta-Mannan et al., 2016). Here use of clodronate to deplete macrophages did not have a great effect on antibody clearance, suggesting that the contribution of macrophages was marginal. This was confirmed by costaining of therapeutic antibody with markers of LSEC to confirm co-localization (Datta-Mannan et al., 2016) with little staining for the bispecific antibodies observed in macrophages. Studies of humanized mice which express human FcγR and are given a humanized antiplatelet antibody confirm these findings with no major effect after macrophage deletion (Schwab et al., 2015). Clearance of opsonized pathogen too is linked to intact FcγRIIb function on LSEC, with deficient mice exhibiting slower pathogen clearance (Ganesan et al., 2012). A more interesting question, however, is what impact LSEC have on the pharmacokinetics and pharmacodynamics of therapeutic antibodies? Also, whether newer antibody formulations can be optimized to exhibit the most favorable dosing profiles and minimize side effects by consideration of LSEC function in health and disease?

### Does Liver Sinusoidal Endothelial Cells Biology Influence the Outcome of Therapeutic Antibody Administration, and Is This Important When Designing Antibodies?

Evidence cited above from knockout animals which have modified hepatic FcR expression confirm the contribution of the liver to clearance. Therapeutic antibody development approaches may include engineering of the Fc portion of humanized antibodies to enhance interactions with FcRn and improve pharmacokinetics. Fc receptor mediated clearance of immune complex is often a desirable therapeutic strategy. Here cell surface Fc receptors bind to the Fc portion of IgG antibodies in immune complexes with their target, and these are cleared from the circulation through uptake into macrophages and endothelial cells in the liver (Lovdal et al., 2000; Ganesan et al., 2012). However, in some situations internalization of therapeutic antibodies *via* actions of FcγRIIb can reduce clinical efficacy, as has been reported for the use of rituximab in some leukemias (Lim et al., 2011) and cancer models (Clynes et al., 2000). It is also noteworthy that circulating immune complex clearance can cause tissue damage and inflammation in some conditions (Johansson et al., 2000). This may be particularly important when the load of circulating IgG is high (Johansson et al., 2000). For example, studies of Humanized DR-5 antibodies (an apoptosis inducing TNFR) with an engineered Fc fragment to enhance FcγRIIb binding in mice engineered to express human FcγRIIb, resulted in increased ALT/AST and mortality (Li and Ravetch, 2012) at supraphysiological doses. Here the FcR was important for the hepatotoxicity. In other studies, humanized antibody designed to target tumor cells by binding to a TNFR stimulatory receptor (CD137) on immune cells to promote anti-tumor immunity responses (Qi et al., 2019) such as Urlumab (Segal et al., 2017) was also associated with liver toxicity, inflammation and liver related adverse events. Mechanistic studies on such antibodies suggest that LSEC expression of FcγRIIb increases crosslinking and activatory effects of strong agonistic antibodies to enhance liver toxicity (Qi et al., 2019). However, engineering of Fab fragments that retain strong agonism minimizes this effect. It is also important to consider potential target-related toxicities alongside FcR-related hepatotoxicity in some cases. As an example, antibodies against TNF were tested as potential anti-inflammatory therapies in human alcoholic hepatitis but some studies were terminated due to adverse outcomes (Blendis and Dotan, 2004) or showed no mortality benefit over standard therapies. There are reports of drug induced toxicity associated with many formulations of anti-TNF antibodies (Lopetuso et al., 2018), particularly in patients with autoimmune liver disease (Tobon et al., 2007) and thus vasculotoxicity associated with antibody clearance could explain an underlying mechanism of damage. However, it is also important to note that TNFα plays a key role in hepatocyte regeneration (Fausto, 2000) and promotes hepatic infiltration by immune cells which drive repair (Chauhan et al., 2020) or fight sepsis which is a significant risk in alcoholic hepatitis (Sharma et al., 2009). Thus biological inhibition of hepatic repair mechanisms may also explain some of the adverse outcomes associated with this approach.

GSK305002 is a humanized IgG antibody that neutralizes the soluble chemokine CCL20 and was in development as a potential therapy for inflammatory disease (Laffan et al., 2020). Although no safety signatures appeared in a phase 1 study in humans, subsequent longer term escalating dose toxicity studies in cynomolgus monkeys highlighted a significant vascular inflammation in most subjects which is unexpected for an antibody targeting soluble antigen. In the liver this presented as moderate inflammation with immune deposits localized within the sinusoids. Target antigen did not appear to be contained in these deposits and importantly anti-human antibodies were not detected or were present at a level too low to explain the findings (Laffan et al., 2020). This would suggest that localization of FcR [or CCL20 (Shields et al., 1999)] on the LSEC may have provided a focus for immune complex deposition and complement mediated toxicity toward the LSEC. Vasculotoxicity has also been seen with other antibody drugs and can present as Sinusoidal Obstruction Syndrome (Jain and Litzow, 2018). This is damage to the sinusoidal endothelium, particularly in central areas of the lobule which exposes the subendothelial cells to blood constituents driving a necrotic response and vascular occlusion. This may relate to drug conjugates bound to antibodies to facilitate target cell toxicity (e.g., calicheamicin for inotuzumab and gemtuzumab). Perhaps the best example of a serious adverse reaction to antibody therapy, the first human trials of the CD28 specific TGN1412 (Suntharalingam et al., 2006) also highlights how important FcR binding is and how hard responses are to predict. TGN1412 is a potent agonistic antibody developed for use in treatment of some cancers and rheumatoid arthritis. Its agonistic events are potentiated by interactions with FcγRIIb, particularly that expressed in B cells (Dudek et al., 2019), but presence of endothelial cells is necessary to recreate the immune activatory responses in *in vitro* assays (Dhir et al., 2012).

Immune or toxic responses to biotherapeutics are complex and can be target related or influenced by the structure and clearance of the antibody itself. For this reason, all new therapeutics are tested extensively in preclinical models and healthy volunteers before proof of efficacy in a patient. However, there are still instances where preclinical models have failed to accurately predict human responses or those in a specific patient cohort or requirements for alternate dosing regimens in chronic disease. Hepatic impairment and impact on antibody kinetics may alter exposure, tolerability and effectiveness if metabolism or excretion is altered (Sun et al., 2020). This may relate to lower albumin production by a damaged liver impacting on antibody exposure of factors which alter expression or function of FcRn and FcγRs could also alter systemic exposure. However, regulatory bodies in some cases suggest that validation of MAb therapy in populations with renal or hepatic impairment is not vital for licensing (Lucas et al., 2018). Moreover, there are clear examples where prior liver injury or older age increase the risk of adverse events of antibody-based treatments (Jain and Litzow, 2018). This has meant that for some antibody-based therapies where hepatotoxic side effects have been noted, pre-existing clinical liver disease is considered an exclusion for use. For example - tocilizumab (humanized IL-6 receptor antibody) and anakinra (IL-1R antagonist antibody) used as anti-inflammatories in rheumatoid arthritis have potential, well described hepatotoxic consequences in some patients (Mahamid et al., 2011) particularly if other immunosuppressive drugs such as methotrexate have been administered.

The challenge remains being able to predict and explain such toxicities, and then to be able to engineer a solution to them. It is important to note that the FcγRs are slightly different in mice (Schwab et al., 2015) than humans and thus variations in human receptors not represented in mice can mean that rodent models are not perfect for predicting humanized antibody activity and clearance. Similarly, IgG4 mAbs don’t interact with monkey FcR’s and thus wouldn’t be picked up in species specific screens (Hansel et al., 2010). Even in a human context, individuals have polymorphisms in Fc: FcR interactions which underpin interindividual variation in antibody clearance and efficacy (Hansel et al., 2010). Levels of FcR expression change with age and disease state. We note above that FcRn expression within the liver is altered in cirrhosis and suggested this could relate to circulating antibody concentration fluctuations in disease (Holdstock et al., 1982) which is clearly associated with poor prognosis (Cacciola et al., 2018). However, it may also be a consequence of age or disease related sinusoidal capillarization (Figure 3). Importantly not all scavenger receptors on LSEC decrease with aging or capillarization. Thus whilst receptors such as CD36 are increased on LSEC with age or development of fatty liver disease (Sheedfar et al., 2014), expression of mannose receptor decreases (Dini et al., 1990) and studies in rats suggest Stabilin-1 and -2 are broadly similar in young and old animals (Simon-Santamaria et al., 2010). Nevertheless, decline in fenestration with age can reduce clearance of drugs such as paracetamol (Mitchell et al., 2011). Similarly, clearance of gut derived LPS is impaired in cirrhosis due to reduced sinusoidal permeability leading to hyperactivation of plasma cells and increased immunoglobulin production (Liu et al., 2015). Capillarization of LSEC also restrict access to hepatocyte FcRn which normally transports antibody across epithelial barriers and maintains circulating antibody concentration (Yeung et al., 2009). Mice that are deficient in FcRn have reduced half-life of administered antibodies (Israel et al., 1996). Coupled with reduced expression of scavenger receptors such as DC-SIGN and FcγR on diseased LSEC this could profoundly alter antibody clearance kinetics. Similarly, occupancy of DC-SIGN by ligands such as viral and bacterial antigens (Gupta and Gupta, 2012) during infection could alter availability for binding antibody-based therapies. In situations of hepatic autoimmunity or disease, clearance of autoantibodies could be managed using FcRn blockers to enhance IgG degradation to manage autoantibodies or control clearance of therapeutic immunoglobulins (Vaccaro et al., 2005). Alternately specific engineering of monoclonal or bispecific antibodies to modify interactions with FcRn could also be used to improve pharmacokinetics (Schutten et al., 1993; Datta-Mannan et al., 2007; Lucas et al., 2018; Datta-Mannan, 2019). This may be particularly important in the context of treating chronic disease if an antibody-based therapy needs to be maintained at therapeutic levels for a long time. Indeed, anti-FcγRIIb antibodies have been suggested as a strategy to reduce clearance of therapeutic antibodies for prolonged administration. However, these were rapidly cleared from the circulation since FcγRIIb is rapidly internalized once antibody binds (Williams et al., 2013). Nevertheless, it is clear that new approaches to antibody design are increasing our abilities to control the pharmacokinetics and targeting of therapeutic antibodies to maximize efficacy whilst minimizing off target effects. In conclusion we have highlighted the often-underestimated role of the liver sinusoidal endothelial cell to antibody clearance. We have also suggested how understanding the changing nature of LSEC in health and disease may explain variations in pharmacokinetics and toxicity in different populations and preclinical models. Challenges to antibody discovery programs are summarized in Table 1. Thus, it seems vital to ensure that future drug development pathways incorporate testing in models with truly representative features and cellular constituents to address issues of poor kinetics, unexpected toxicity and poor predictive ability.

**TABLE 1:** Clinical challenges associated with hepatic clearance of biological therapies and strategies to mitigate risk during drug development.

Clinical challenge	Explanation	Mitigating strategy
Impact of LSEC Fc receptors on antibody PK	Accelerated or delayed clearance of circulating antibody	Modify Fc portion to enhance interaction with FcRn and improve half life Modify Fc portion to minimize interaction with FcγRIIb
Localized hepatotoxicity or DILI in reponse to antibody therapy in humans	Enhanced deposition and clearance by LSEC leading to vasculotoxicity	Analysis of Fc portion and specific testing of clearance by human FcR to minimize crosslinking and activation in sinusoid
Complement mediated toxicity/Sinusoidal obstruction syndrome associated with antibody therapy	Immune complex binding to LSEC and cell apoptosis leading to exposure of basal lamina	Careful screening for binding to Fc receptors on LSEC
Altered antibody PK in older patients or patients with underlying liver disease	LSEC capillarization, reduction in hepatic albumin production	Careful screening for pre-existing disease in patient populations. Age-dependent pharmacokinetic assessment at Phase 1 testing
Complications due to autoantibody production in hepatic autoimmunity	LSEC capillarization or autoantibody occupancy of FcRs impacting on PK	Use of FcRn blockers to enhance IgG degradation
Desire to improve half life of therapeutic antibody	Accelerated clearance by hepatic FcγRIIb	Engineering of Fc portion to minimize interaction or delay internalization of receptor
Lack of clinical efficacy upon testing in human subjects	Reduced abilities of rodent or primate models to recreate human hepatic antibody clearance	Inclusion of human cell based or tissue array screens in pre-trail development stages

## Author Contributions

All authors listed have made a substantial, direct, and intellectual contribution to the work, and approved it for publication.

## Conflict of Interest

The authors declare that the research was conducted in the absence of any commercial or financial relationships that could be construed as a potential conflict of interest.

## Publisher’s Note

All claims expressed in this article are solely those of the authors and do not necessarily represent those of their affiliated organizations, or those of the publisher, the editors and the reviewers. Any product that may be evaluated in this article, or claim that may be made by its manufacturer, is not guaranteed or endorsed by the publisher.
